# Assessment of pain associated with bovine respiratory disease and its mitigation with flunixin meglumine in cattle with induced bacterial pneumonia

**DOI:** 10.1093/jas/skab373

**Published:** 2021-12-21

**Authors:** Miriam S Martin, Michael D Kleinhenz, Brad J White, Blaine T Johnson, Shawnee R Montgomery, Andrew K Curtis, Mikaela M Weeder, Dale A Blasi, Kelli M Almes, Raghu G Amachawadi, Harith M Salih, Matt D Miesner, Angela K Baysinger, Jason S Nickell, Johann F Coetzee

**Affiliations:** 1 Department of Anatomy and Physiology, Kansas State University College of Veterinary Medicine, Manhattan, KS 66506; 2 Department of Clinical Sciences, Kansas State University College of Veterinary Medicine, Manhattan, KS 66506; 3 Department of Diagnostic Medicine and Pathobiology & Kansas State Veterinary Diagnostic Laboratory, Kansas State University College of Veterinary Medicine, Manhattan, KS 66506; 4 Department of Animal Science, Kansas State University, Manhattan, KS 66506; 5 Merck Animal Health, De Soto, KS 66018

**Keywords:** BRD, pain, biomarkers, lung lesion scores

## Abstract

Pleuritic chest pain from bacterial pneumonia is often reported in human medicine. However, studies investigating pain associated with bovine respiratory disease (**BRD**) are lacking. The objectives of this study were to assess if bacterial pneumonia elicits a pain response in calves with experimentally induced BRD and to determine the analgesic effects of transdermally administered flunixin. A total of 26 calves, 6–7 mo of age, with no history of BRD were enrolled into one of three treatment groups: 1) experimentally induced BRD + transdermal flunixin at 3.3 mg/kg twice, 24 h apart (BRD + FTD); 2) experimentally induced BRD + placebo (BRD + PLBO); and 3) sham induction + placebo (CNTL + PLBO). Calves induced with BRD were inoculated with *Mannheimia haemolytica* via bronchoalveolar lavage. Outcomes were collected from −48 to 192 h post-treatment and included serum cortisol, infrared thermography, mechanical nociceptive threshold, substance P, kinematic gait analysis, visual analog scale (**VAS**), clinical illness score, computerized lung score, average activity and rumination level, prostaglandin E_2_ metabolite, plasma serum amyloid A, and rectal temperature. Outcomes were evaluated using either a generalized logistic mixed model for categorical variables or a generalized linear mixed model for continuous variables. Right front force differed by treatment (*P* = 0.01). The BRD + PLBO had lower mean force applied to the right front limb (85.5 kg) compared with BRD + FTD (96.5 kg; *P* < 0.01). Average VAS differed by a treatment by time interaction (*P* = 0.01). The VAS scores differed for BRD + PLBO at −48 (3.49 mm) compared with 168 and 192 h (13.49 and 13.64 mm, respectively) (*P* < 0.01). Activity for BRD + PLBO was higher at −48 h (27 min/h) compared with 48, 72, 120, and 168 h (≤ 22.24 min/h; *P* < 0.01). Activity differed by a treatment by time interaction (*P* = 0.01). Activity for BRD + FTD was higher at −48 and 0 h (28.2 and 28.2 min/h, respectively) compared to 48, 72, 96, and 168 h (≤23.7 min/h; *P* < 0.01). Results show a combination of reduced activity levels, decreased force on the right front limb, and increased VAS pain scores all support that bacterial pneumonia in cattle is painful. Differences in right front force indicate that flunixin transdermal may attenuate certain pain biomarkers in cattle with BRD. These findings suggest that BRD is painful and analgesic drugs may improve the humane aspects of care for cattle with BRD.

## Introduction

Bovine respiratory disease (**BRD**) is the most common and costliest disease affecting the beef industry (DeDonder et al., 2010; Peel, 2020), with BRD death loss alone costing the beef industry upwards of $907 million annually in the USA (USDA, 2017). The most commonly isolated organism from BRD-affected lung tissue in feedyard settings is an opportunistic pathogen, *Mannheimia haemolytica* (Griffin et al., 2010). *M. haemolytica* is considered a commensal organism that, when cattle experience stress, can proliferate into the nasopharynx and translocate to the lung, where it causes fibrinous pleuropneumonia (Rice et al., 2007). Signs of BRD include nasal and ocular discharge, depression, anorexia, and increased respiratory rate with dyspnea. Clinical outcomes may range from hardly noticeable to death (Griffin et al., 2010). Pleuritic chest pain resulting from bacterial pneumonia is commonly reported in human medicine (Boyd et al., 2006). However, published literature regarding the association of pain and respiratory disease in cattle is lacking.

Cattle are stoic animals by nature and have long been subject to evolutionary pressure to mask pain (Hudson et al., 2008). Further research into the development of appropriate behavioral and physiological pain assessment tools is needed to objectively quantify pain and search for the most effective analgesic strategies to mitigate pain (Schwartzkopf-Genswein et al., 2012). An analgesic class commonly employed in food animals to reduce pain are nonsteroidal anti-inflammatory drugs (**NSAIDs**). This class inhibits the cyclooxygenase enzymes, reduces inflammation, and decreases prostaglandin production, which attenuates the response of the peripheral and central components of the nervous system to noxious stimuli, and results in a reduction in response to pain (Ochroch et al., 2003). The objectives of this study were to assess if bacterial pneumonia elicits a pain response in calves with experimentally induced BRD and to determine the analgesic effects of flunixin meglumine transdermal in experimentally induced bacterial pneumonia.

## Materials and Methods

### Animals, housing, and treatments

The Institutional Animal Care and Use Committee and the Institutional Biosafety Committee of Kansas State University reviewed and approved the experimental protocol for this project (IACUC# 4465 and IBC# 1499). This study was conducted at the Kansas State University Stocker Unit near Manhattan, KS, between October and December 2020. A total of 50 weaned and vaccinated male Holstein calves were received for potential enrollment in the study in June 2020. Calves were vaccinated 2 wk prior to delivery against disease caused by infectious bovine rhinotracheitis, bovine virus diarrhea (**BVD** Type 1 and BVD Type 2), parainfluenza Type 3, and bovine respiratory syncytial viruses (Vira Shield 6; Elanco, Greenfield, IN; BOVILIS Nasalgen 3; Merck Animal Health, Kenilworth, NJ), to aid in the prevention of diseases caused by *Clostridium chauvoei*, *Clostridium septicum*, *Clostridium novyi* Type B, *Clostridium haemolyticum* (known also as *C. novyi* Type D), *Clostridium tetani*, and *Clostridium perfringens* Types C and D (COVEXIN 8; Merck Animal Health), and against endotoxin-mediated diseases caused by *Escherichia coli*, *Salmonella typhimurium*, *Pasturella multocida, M. haemolytica*, and mastitis due to *E. coli* (ENDOVAC – Dairy; Endovac Animal Health, Columbia, MO).

Calves were dehorned at 2 mo and castrated at 4 mo of age at Kansas State University.

Calves were housed in two outdoor pens with dirt flooring of equal size with 13 calves/pen. Once inoculated, calves were housed in a pen with solid paneling around the perimeter to prevent calves from touching noses with calves not currently on the study. Diets were formulated to meet or exceed the nutritional requirements set by the National Research Council (2000) at 2.2% BW and all calves were fed once daily per normal procedures at the study site. The diet was formulated to contain 39.5% corn, 40% sweet bran, 13% prairie hay, and 7.5% supplement (wheat midds, calcium, salt, soybean oil, monensin, and vitamins) all on a dry matter basis. Calves were moved to the study site 2 wk prior to the study start date to allow for an acclimation period. Upon arrival to the study site, calves were affixed with a 3-axis accelerometer ear-tag (Allflex Livestock Intelligence, Madison, WI) to quantify activity and daily rumination time.

**Figure 1.**
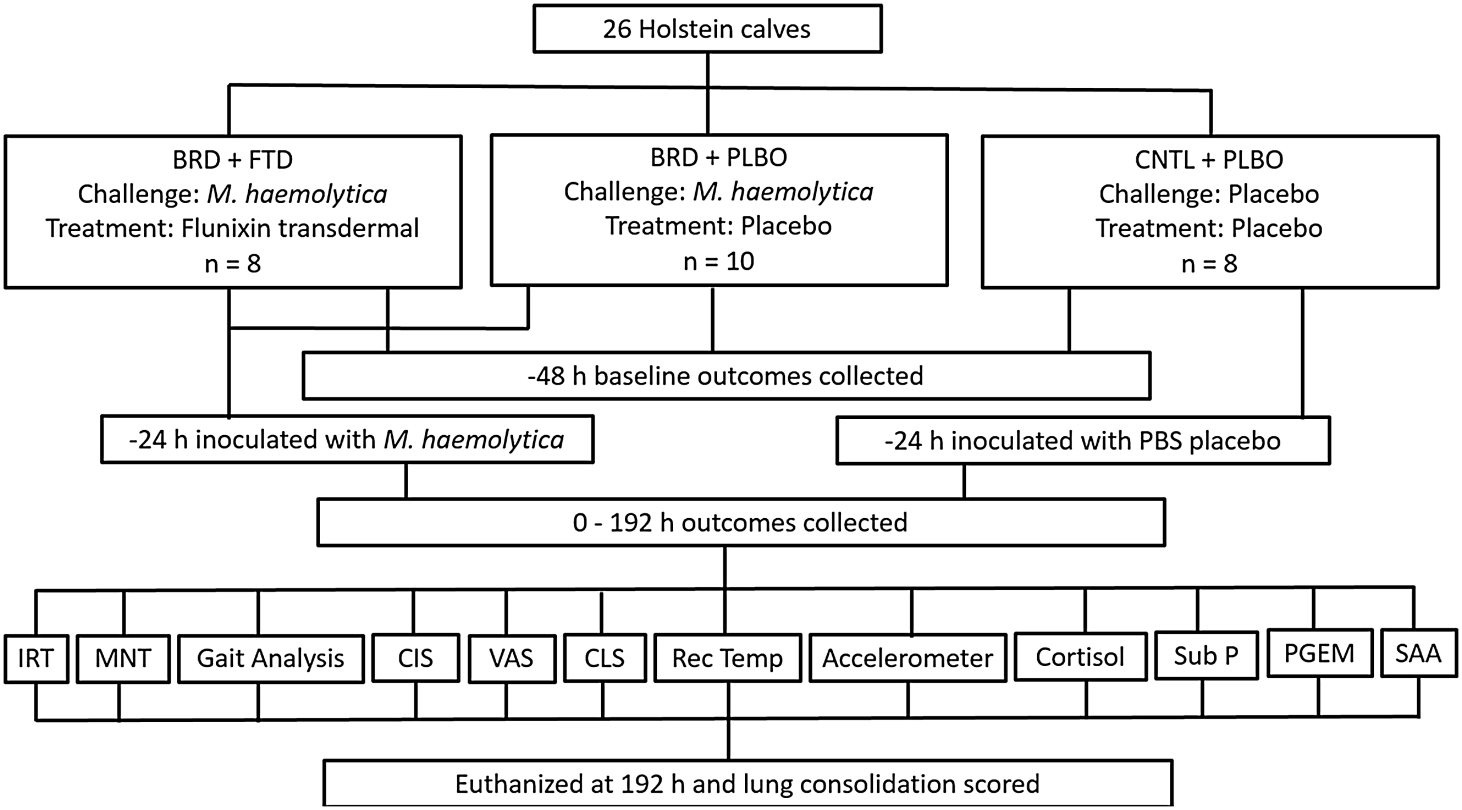
Flow chart outlining the timing of study events.

A total of 26 calves, 6–7 mo of age, weighing an average of 185 ± 4 kg were then enrolled onto the study based on the following inclusion criteria: 1) healed from dehorning and castration procedures, 2) negative for lung pathology on thoracic ultrasound as described by Ollivett and Buczinski (2016), 3) negative bovine viral diarrhea test (IDEXX Laboratories, Inc., Westbrook, ME), 4) no history of clinical signs of BRD while at Kansas State University following weaning, and 5) upon physical exam no signs of illness, lameness, or joint swelling. After the acclimation period, prior to the start of the study, calves were weighed and randomly allocated to one of three experimental treatment groups (Figure 1). Cattle were uniquely identified with individual ear tags prior to acclimation period and randomized utilizing the RAND function in Microsoft Excel (Microsoft Excel 2016, Microsoft Corporation, Redmond, WA). The treatment groups were as follows: (BRD + FTD) − experimentally induced respiratory disease + transdermal flunixin administered topically at 3.3 mg/kg twice, 24 h apart, (BRD + PLBO) − experimentally induced respiratory disease + placebo, (CNTL + PLBO) − sham induction + placebo. Eight calves were assigned to each of the BRD + FTD and CNTL + PLBO groups and 10 calves were assigned to the BRD + PLBO group due to the higher risk of calves reaching the endpoint criteria in that treatment group. Calf was the experimental unit for the study. The study took place in two phases, with 13 calves in each phase, with each phase being housed in one pen. The phases were put into place due to the terminal nature of the study and the constraints of the veterinary diagnostic laboratory necropsy capacity. The study duration was 9 d/phase from calves being enrolled onto the study to the completion of the study phase.

At 48 h prior to the start of the study, baseline parameters were collected and IceTag (IceRobotics Ltd, South Queensferry, Edinburgh, Scotland, UK) accelerometers were placed on the left rear leg of each calf for the duration of the study. At 24 h prior to the start of the study, calves in the BRD + FTD and BRD + PLBO treatment groups were inoculated with a strain of *M. haemolytica* obtained from a USDA research facility (U.S Meat Animal Research Center [MARC], Clay Center, NE) using bronchoalveolar lavage (**BAL**) as described by Theurer et al. (2013). The strain was characterized by the USDA MARC facility as being serotype A1 and susceptible to the macrolide class of antimicrobials. The right apical lung lobes were inoculated using broncho-selective endoscopy. A 10 mL dose of *M. haemolytica* at 1 × 10^9^ cfu/mL was used to inoculate each calf, and then the endoscope was flushed with 60 mL of the phosphate-buffered solution to achieve a total volume of 70 mL. The CNTL + PLBO calves received 70 mL of phosphate-buffered solution as described earlier.

After inoculation, calves were monitored for disease onset using visual clinical illness scoring conducted by two trained evaluators blinded to treatment every 6 h as previously described in Perino and Apley (1998). Trial-by-trial interobserver agreement was 78%. Clinical signs of illness included lethargy, anorexia, nasal or ocular discharge, tachypnea, and fever (rectal temperature > 40 °C). At 24 h post-inoculation when all calves inoculated with *M. haemolytica* were exhibiting clinical signs of BRD, calves received one of the following treatments: Topical flunixin meglumine (Banamine Transdermal, Merck Animal Health, Madison, NJ) at 3.33 mg/kg (1 mL/15 kg) or topical placebo at 1 mL/15 kg. The placebo was made up of propylene glycol, isopropyl alcohol, and a red dye to mimic the test product in color, viscosity, and odor, as described in Kleinhenz et al. (2017). The flunixin transdermal and placebo were administered on dry skin. The entire dose was applied on the dorsal midline between the withers and tail head in accordance with label directions. The time of treatment was considered time 0 h for the study. Calves with a fever (rectal temperature > 40 °C) at any point in time throughout the study were administered a single subcutaneous injection of tildipirosin (Zuprevo 18%; Merck Animal Health, Madison, NJ) at a dose of 4 mg/kg.

### Measurements and sample collection

Outcome variables were collected at −48, 0, 4, 8, 24, 32, 48, 72, 96, 120, 144, 168, and 192 h post-treatment (Figure 1), in addition to the 3-axis accelerometer ear-tags and accelerometers continuously collecting activity and rumination data throughout the study. Outcome variables collected at the given timepoints included rectal temperature, infrared thermography (**IRT**) imaging, kinematic gait analysis, mechanical nociception threshold (**MNT**), visual analog scale (**VAS**) score, clinical illness score (**CIS**), computerized stethoscope (Whisper Veterinary Stethoscope, Merck Animal Health, Madison, NJ) lung score (**CLS**), and blood sampling for serum cortisol, substance P, prostaglandin E_2_ metabolite (**PGEM**), and serum amyloid A (**SAA**) analysis. All trained evaluators were blinded to treatment for the duration of the study. Two blinded evaluators scored CIS and VAS, one blinded graduate student operated the computerized stethoscope. Following the 192 h collection, calves were euthanized and transported to the Kansas State Veterinary Diagnostic Laboratory for necropsy and lung lesion scoring.

The IRT images captured the medial canthus of the left eye using a research-grade infrared camera (Fluke TiX580, Fluke Corp, Everett, WA) as described in Martin et al. (2021). Infrared images were analyzed using research-specific computer software (SmartView v. 4.3, Fluke Thermography, Plymouth, MN) to determine maximum and minimum temperatures. The difference between the temperature of the medial canthus baseline and the timepoints following were determined and used for statistical analysis.

A commercially available pressure mat kinematic gait analysis system (Walkway, Tekscan Inc., South Boston, MA) was used to record gait and biomechanical parameters as described in Martin et al. (2021). The pressure mat was calibrated using a known mass to ensure the accuracy of the measurements at each timepoint. Video synchronization was used to ensure consistent gait between and within calves at each timepoint. Using research-specific software (Walkway 7.7, Tekscan Inc.), force, contact pressure, impulse, stance time, and stride length were assessed.

A hand-held pressure algometer (Wagner Instruments, Greenwich, CT) was used for MNT determination. A force was applied perpendicularly, at a rate of approximately 1 kg of force per second, at 1 location on both the left and right side of the ribs of each calf, over the 6th intercostal space for a total of 2 locations, as described in Williams et al., 2020). A withdrawal response was indicated by an overt movement away from the applied pressure algometer. Locations were tested three times in sequential order, and the values were averaged for statistical analysis. A second investigator recorded the MNT values to prevent bias by the investigator performing the MNT collection. The collection of MNT values was recorded at only −48, 8, 24, 72, and 192 h timepoints to prevent sensitization of the calf.

A VAS score was assigned by two trained evaluators blinded to treatment allocations using methods adapted from Martin et al. (2020). The VAS used was a 100 mm (10 cm) line anchored at each end by descriptors of “No Pain” or “Severe Pain.” Seven parameters were used to assess pain: depression, tail swishing or flicking, stance, head carriage, spinal alignment, movement, and ear carriage also adapted from Martin et al. (2020). No pain was characterized by being alert and quick to show interest, no tail swishing, a normal stance, head carriage above spine level, a straight spine, moving freely around the pen and ears forward. Severe pain was characterized by being dull and showing no interest, more than three tail swishes per minute, legs abducted, head held below spine level, a curved spine, reluctant to move, and ears down. The evaluator marked the line between the two descriptors to indicate the pain intensity. A millimeter scale was used to measure the score from the zero anchor point to the evaluator’s mark. The mean VAS scores of the two evaluators were combined into one score for statistical analysis.

A CIS was assigned by two trained evaluators blinded to treatment allocations. The CIS consisted of: 1) is a normal healthy animal, 2) slightly ill with mild depression or gauntness, 3) moderately ill demonstrating severe depression/labored breathing/nasal or ocular discharge, and 4) severely ill and near death showing minimal response to human approach (Perino and Apley, 1998). The CIS scores of the two evaluators were combined into one score for statistical analysis, if either evaluator scored >1 then a final score >1 was assigned, with 1 being considered normal and greater than 1 considered abnormal.

A computerized stethoscope (Whisper, Merck Animal Health, De Soto, KS) was used to analyze lung and heart sounds via a machine-learning algorithm that assigns a CLS from 1 to 5, with 1 being normal and 5 being severely compromised lung tissue (Nickell et al., 2020). The bell of the lung stethoscope was placed approximately two inches caudal and dorsal to the right elbow of each calf, and lung sounds were recorded for 8 s. If the recording was deemed acceptable by the computer program, the score was recorded, if not another recording was taken.

Calves were affixed with a 3-axis accelerometer ear-tag (Allflex Livestock Intelligence, Madison, WI) to quantify activity and daily rumination time throughout the study. The average number of minutes spent active or ruminating over 60 min time periods for the study duration was then calculated. An IceTag (IceRobotics Ltd, South Queensferry, Edinburgh, Scotland, UK) accelerometer was also placed on the left rear leg of each calf for the duration of the study. Accelerometer ID was paired with calf ID prior to placement onto the calf. Accelerometers were removed at the conclusion of the study and data were downloaded from the accelerometers for analysis. Steps, standing, lying, lying bouts, and motion index data were collected via the accelerometers and analyzed as described in Martin et al. (2020).

Rectal temperatures were taken daily by placing a digital thermometer (180 Innovations, Lakewood, CO) against the rectum wall until a temperature reading was produced on the thermometer’s screen.

Blood samples for serum cortisol, substance P, PGEM, and SAA determination were collected from the jugular vein via venipuncture. The whole blood samples were immediately transferred to tubes (Vacutainer, BD Diagnostics, Franklin Lakes, NJ) containing either no additive for cortisol determination or EDTA anticoagulant for PGEM determination. For substance P determination, benzamidine hydrochloride (final concentration of 1mM) was added to EDTA blood tubes 48 h prior to collection. Samples were immediately placed on ice after collection, centrifuged within 30 min of collection for 10 min at 1,500 × *g*, and serum and plasma were placed in cryovials via transfer pipette and stored at −80 °C.

Serum cortisol concentrations were determined using a commercially available radioimmunoassay (**RIA**) kit (MP Biomedicals, Irvine, CA) following manufacturer specifications with minor modifications as described in Martin et al. (2021); the standard curve was extended to include 1 and 3 ng/mL by diluting the 10 and 30 ng/mL manufacturer-supplied standards, 1:10, respectively. The standard curve ranged from 1 to 300 ng/mL. A low (25 ng/mL) and high (150 ng/mL) quality control (**QC**) were ran at the beginning and end of each set to determine inter-assay variability. Plain 12 × 75 mm polypropylene tubes were used as blank tubes to calculate non-specific binding. Input for standards, QCs, and samples was adjusted to 50 µL. Samples were incubated at room temperature for 30 min prior to the addition of I-125. Manufacturer instructions were then followed. Tubes were counted on a gamma counter (Wizard2, PerkinElmer, Waltham, MA) for 1 min. The raw data file was then uploaded onto MyAssays Desktop software (version 7.0.211.1238, 21 Hampton Place, Brighton, UK) for concentration determination. Standard curves were plotted as a 4-parameter logistic curve. Samples with a coefficient of variation (**CV**) >18% were reanalyzed.

Substance P (SP) concentrations were determined through RIA using methods described by Van Engen et al. (2014). The standard curve, ranging from 20 to 1,280 pg/mL, was created by diluting synthetic SP (Phoenix Pharmaceuticals, Burlingame, CA) with RIA Buffer (50 mM sodium phosphate dibasic heptahydrate, 13 mM disodium EDTA, 150 mM sodium chloride, 1 mM benzamidine hydrochloride, 0.1% gelatin, 0.02% sodium azide; pH 7.4). A 100 µL of samples, standards, and QCs were aliquoted into plain 12 × 75 mm conical bottom tubes followed by 100 µL of Rabbit anti-SP primary antibody (1:20,000; Phoenix Pharmaceuticals). Iodine-125-SP tracer (custom iodination by PerkinElmer) was diluted with RIA buffer to 20,000 cpm, then 100 µL was added to the samples, standards, and QCs. Samples were then covered and stored at 4 °C for 48 h. At the end of the 48 h incubation, samples were placed on ice and 100 µL of normal rabbit plasma (1:80) and goat anti-rabbit secondary antibody (1:40; Jackson ImmunoResearch, West Grove, PA) were added to each tube. Samples were then incubated at room temperature for 10 min, placed back on ice, and 100 µL of blank bovine plasma was added to the standards and QCs. All tubes then had 1 mL of 12% polypropylene glycol in 0.85% sodium chloride added. Samples were centrifuged at 3,000 × *g* for 30 min at 4 °C and the supernatant aspirated. Tubes were counted on a gamma counter (Wizard2, PerkinElmer, Waltham, MA) for 1 min. The raw data file was then uploaded onto MyAssays Desktop software for concentration determination. Standard curves were plotted as a 4-parameter logistic curve. Samples with a CV >18% were reanalyzed.

PGEM were analyzed using a commercially available ELISA kit (Cayman Chemical, Ann Arbor, MI) following manufacturer specifications with minor modifications as described in Martin et al. (2021). Sample input was adjusted to 375 µL with 1.5 mL ice-cold acetone added for sample purification. Samples were incubated at −20 °C for 30 min, then centrifuged at 3,000 × *g* for 5 min. The supernatant was transferred to clean 13 × 100 mm glass tubes and evaporated using a CentriVap Concentrator (Labconco, Kansas City, MO) overnight (approx. 18h). Samples were reconstituted with 375 µL of appropriate kit buffer. A 300 µL aliquot of the reconstituted sample was derivatized with proportionally adjusted kit components. Manufacturer protocol was then followed. Samples were diluted at 1:2 and ran in duplicate. Absorbance was measured at 405 nm after 60 min of development (SpectraMax i3, Molecular Devices, San Jose, CA). Sample results were excluded if the raw read exceeded the raw read of the highest standard (Standard 1; 50 pg/mL) or was below the lowest acceptable standard. The lowest acceptable standard was defined for each individual plate and was identified by excluding standards that had a ratio of absorbance of that standard to the maximum binding of any well (%B/B_0_) of ≥80% or ≤20%. Any individual sample outside the standard curve, with a %B/B_0_ outside the 20%–80% range, or a CV >15% were reanalyzed. PGEM were analyzed at −48, 0, 72, and 192 h timepoints.

SAA concentrations were determined in serum samples using an ELISA assay (Phase Range Multispecies SAA ELISA kit; Tridelta Development Ltd, Maynooth, Kildare, Ireland). Manufacturer specifications were followed and samples were diluted as necessary. Absorbance was measured at 450 nm on a SpectraMax i3 plate reader (Molecular Devices). Raw data were analyzed using MyAssays Desktop software for concentration determination. Standard curves were plotted as a 4-parameter logistic curve. Samples with a CV >15% were reanalyzed.

Flunixin (**FLU**; Sigma Aldrich, St. Louis, MO) and flunixin-d3 (**FLU-d3**, internal standard, Toronto Research Chemicals, North York, ON, Canada) stock solutions were prepared at 1 mg/mL in methanol and acetonitrile respectively and stored at −80 °C. FLU standard curve and QCs were prepared fresh daily in negative control plasma. The standard curve ranged from 1 to 100 ng/mL. A 50 ng/mL working solution of FLU-d3 was prepared daily by diluting the 1 mg/mL stock in 0.1% formic acid in acetonitrile. Plasma collected in lithium heparin tubes was used for flunixin concentration determination. Samples were extracted via protein precipitation. Briefly, 100 µL of sample, standards, QCs, and blanks were aliquoted and 400 µL of 50 ng/mL FLU-d3 in 0.1% formic acid in acetonitrile was added. Samples were then centrifuged at 3,000 × *g* for 5 min. Supernatant was decanted into 75 × 100 mm glass tubes, evaporated using a CentriVap system (Labconco), and reconstituted with 200 µL of 25% acetonitrile in water. The reconstituted samples were transferred to clean microcentrifuge tubes and centrifuged at 10,000 × *g* for 7 min. An aliquot of 100 µL was transferred to an autosampler vial with a glass insert (QsertVial, Waters Corp., Milford, MA) with pre-slit septum lids. Vials were loaded onto an Acquity H Class ultra-performance liquid chromatography (**UPLC**) system coupled with a Xevo TQ-S tandem mass spectrometer (MS/MS; Waters Corp.). Chromatographic separation was achieved using an Aquity UPLC BEH C18 column held at 40 °C during analysis. Mobile phase A and B consisted of 0.1% formic acid in acetonitrile and 0.1% formic acid in 18.2 MΩ.cm water, respectively. Flow rate was set at 0.4 mL/min with the following gradient during the 3 min run time: 30% A at 0–1.49 min, 100% A from 1.5 to 2 min, then 30% A at 2.01 min. The Xevo TQ-S MS/MS was equipped with an electrospray ionization interface set in positive mode. The quantifying transition for FLU was *m/z* 297.27→279.24 and the qualifying transition was *m/z* 297.27→264.15. The quantifying transition for FLU-d3 was *m/z* 300.23→282.26. Data acquisition and analysis were performed using MassLynx and TargetLynx software, respectively (Waters Corp). The standard curve was linear from 1 to 100 ng/mL and the correlation coefficient was accepted if it was at least 0.975. Samples with flunixin concentrations outside the standard curve linear range were diluted at 1:500 with blank plasma and reanalyzed.

All calves were sedated with intravenous xylazine (0.1 mg/kg) and humanely euthanized following the 192 h timepoint with a penetrating captive bolt stunner (CASH Special, FRONTMATEC Accles & Shelvoke Ltd., Minworth, Sutton Coldfield, UK) followed by intravenous injection of potassium chloride (120 mL). A pathologist (K.M.A.), blinded to treatment groups, performed a postmortem examination to determine lung lesions and assign a lung lesion score based on lung consolidation. The lung lesion score was determined using methods described by Fajt et al. (2003). The equation used was as follows: total percentage lung consolidation = (0.053 × cranial segment of left cranial lobe %) + (0.049 × caudal segment of left cranial lobe %) + (0.319 × left caudal lobe %) + (0.043 × accessory lobe %) + (0.352 × right caudal lobe %) + (0.061 × right middle lobe %) + (0.060 × caudal segment of right cranial lobe %) + (0.063 × cranial segment of right cranial lobe %).

### Calculations and statistical analysis

A sample size calculation was performed a priori using rectal temperature means derived from Theurer et al. (2013) to determine a sample size of eight calves per treatment group. The study was designed to have power exceeding 0.80 assuming a difference in effect size (Δ) of 0.75 °C, a standard error (*σ*) of 0.5, and a statistical inference level (*α*) of 0.05. Study data were imported into a commercially available statistical software package (R: An open-source programming language and environment for statistical computing, version 4.0.5, R Foundation for Statistical Computing, Vienna, Austria) for descriptive analyses and hypotheses testing. Twenty-four-hour accelerometer readings were calculated from 8 a.m. to 8 a.m., coinciding with sample collection timepoints. A scoring system was adapted from White et al. (2012), with the addition of a medium category, to categorize consolidated lung tissue lesion scores as low ≤10%, medium >10% and ≤20%, and high >20%. Treatment for BRD based on pyrexia was dichotomized into a yes/no (0/1) category for analysis. The outcomes for CIS and CLS were dichotomized with a score of 1 considered normal and >1 considered abnormal. Each outcome parameter was evaluated using either a generalized logistic mixed model for categorical variables or a generalized linear mixed model for continuous variables. Packages used for modeling outcomes of interest included lme4, lmerTest, and car (Bates et al., 2015; Kuznetsova et al., 2017; Fox and Weisberg, 2019). The effect of treatment, time and time × treatment interaction were evaluated using a linear mixed model that included the covariates of categorized lung score and dichotomized tildipirosin administration. A random intercept was included to account for lack of independence and hierarchical data structure of calf within study phase. Due to the lack of observations for plasma flunixin levels due to flunixin concentrations only being detected out to the 144 h timepoint, the parameter was removed from models for them to converge. Model estimated means using the emmeans package are reported for continuous variables and probabilities are reported for categorical variables. A Tukey–Kramer familywise error-adjustment was used for multiple comparisons. The Hmisc and ggcorrplot packages were used to evaluate Pearson correlations between outcomes (Kassambara, 2019; Harrell, 2021). Statistical significance was set a priori at *P* ≤ 0.05. The following pharmacokinetic parameters were calculated using non-compartmental methods using PK Solver (Zhang et al., 2010) in Excel: slope of the terminal phase (*λ*_z_), terminal half-life (T½), maximum plasma concentration (C_max_); time to achieve peak concentration (T_max_), area under the curve from the time of dosing (Dosing_time_) to the last measurable (positive) concentration (AUC_0–last_), and AUC from Dosing_time_ extrapolated to infinity, based on the last observed concentration (obs) (AUC_0–∞_).

## Results

Outcome measure means by treatment are outlined in Table 1. Throughout the study duration, five calves received tildipirosin for BRD based on pyrexia (rectal temperature > 40 °C). Two calves who received tildipirosin were in the BRD + FTD group, one calf was in the BRD + PLBO group, and two calves were in the CNTL + PLBO group.

**Table 1.** Least squares means and probabilities ± SEM for outcome variables by treatment group with associated *P*-values^1^

Outcome^2^	Units	Treatment group least squares means			*P*-values		
		BRD + PLBO	BRD + FTD	CNTL + PLBO	Treatment	Timepoint	Treatment × Timepoint
IRT	°C	30.3 ± 0.39	30.5 ± 0.40	30.3 ± 0.41	0.91	<0.01∗	0.99
Rf stance time	s	0.73 ± 0.04	0.72 ± 0.0414	0.85 ± 0.04	0.07	<0.01∗	0.85
Rf stride length	cm	120 ± 3.37	124 ± 3.51	114 ± 3.54	0.11	<0.01∗	0.13
Rf force	kg	85.5^a^ ± 3.29	96.5^b^ ± 3.42	84.8^a,b^ ± 3.48	0.01^∗^	0.01^∗^	0.87
Rf impulse	kg∗s	43.8 ± 3.16	49.3 ± 3.29	50.2 ± 3.34	0.22	0.32	0.66
Rf pressure	kg/cm^2^	5.64 ± 0.19	5.94 ± 0.20	5.81 ± 0.20	0.41	0.01^∗^	0.59
Gait distance	cm	159 ± 3.39	159 ± 3.57	161 ± 3.60	0.86	0.06	0.32
Gait velocity	cm/s				0.004	0.52	0.05^∗^
MNT average	kg F	1.70 ± 0.11	1.77 ± 0.11	1.54 ± 0.12	0.35	<0.01∗	0.14
MNT % change	%	0.06 ± 0.11	0.01 ± 0.11	−0.07 ± 0.11	0.73	0.61	0.68
VAS	1–10 cm				0.45	0.56	0.01^∗^
Average activity	min/h				0.24	0.01	0.01^∗^
Average rumination	min/h	36.6 ± 3.62	38.7 ± 3.75	34.6 ± 3.82	0.73	0.01^∗^	0.50
Motion index	index	5,301 ± 540	5,832 ± 616	5,095 ± 573	0.60	<0.01∗	0.21
Standing time	proportion	0.40 ± 0.02	0.42 ± 0.02	0.42 ± 0.02	0.52	<0.01∗	0.95
Lying time	proportion	0.60 ± 0.02	0.58 ± 0.02	0.58 ± 0.02	0.64	<0.01∗	0.93
Steps	count	1,112 ± 105	1,209 ± 120	1,084 ± 111	0.66	<0.01∗	0.08
Lying bouts	count	13.2 ± 1.20	13.9 ± 1.3	11.7 ± 1.27	0.46	0.03^∗^	0.53
Rectal temperature	°C	39.1 ± 0.14	39.2 ± 0.15	39.2 ± 0.15	0.98	<0.01∗	0.15
Cortisol	ng/mL	4.83 ± 0.79	4.09 ± 0.86	3.22 ± 0.88	0.19	0.01^∗^	0.10
Substance P	pg/mL	359 ± 64.3	361 ± 66.7	216 ± 67.9	0.14	0.07	0.90
PGEM	pg/mL	29.5 ± 4.27	25.8 ± 4.43	32.3 ± 4.51	0.44	< 0.01∗	0.84
SAA	µg/mL	221 ± 54.3	279 ± 56.2	225 ± 57.2	0.59	<0.01	0.24
		Treatment group probabilities of score being abnormal					
		BRD + PLBO	BRD + FTD	CNTL + PLBO			
CIS	Score 1–4	0.156 ± 17.1	0.118 ± 13.4	0.148 ± 16.3	0.96	<0.01∗	0.57
CLS	Score 1–5	0.626 ± 0.08	0.581 ± 0.09	0.565 ± 0.09	0.84	<0.01∗	0.98

Means are not reported for outcomes with significant interactions. ^2^IRT, infrared thermography; Rf, right front; MNT, mechanical nociceptive threshold; VAS, visual analog scale; PGEM, prostaglandin E_2_ metabolites; SAA, serum amyloid A; CIS, clinical illness score; CLS, computerized lung score. ^∗^Statistically significant difference (*P* ≤ 0.05) was observed. ^a,b^Different superscripts indicate significant differences between treatment groups (*P* ≤ 0.05).

### Infrared thermography

IRT average temperature did not differ by treatment (*P* = 0.91), treatment by time interaction (*P* = 0.99), or lung category (*P* = 0.29), but did differ by timepoint (*P* < 0.01). Average temperature readings at 144, 168, and 192 h (27.4, 27.1, and 28.4 °C, respectively) were less than readings from 0 to 96 h (≥30.1 °C; *P* < 0.01). After treatment, the highest readings were at 8 and 32 h (32.9 and 35.3 °C, respectively).

### Kinematic gait analysis

Right front stance time did not differ by treatment (*P* = 0.07), treatment by time interaction (*P* = 0.85), or lung category (*P* = 0.64) but did differ by timepoint (*P* < 0.01). Stance time means were longer at 72 and 96 h (0.916 and 0.905 s, respectively) relative to −48 to 24 h (≤0.728 s) (*P* < 0.03).

Right front stride length did not differ by treatment (*P* = 0.11) or treatment by time interaction (*P* = 0.13) but did differ by timepoint (*P* < 0.01) and lung category (*P* = 0.01). Calves had a shorter right front stride length at 72 and 96 h (114 and 112 cm, respectively) relative to −48 and 32 h (128 and 123 cm, respectively; *P* < 0.05). Calves in the high lung lesion category had shorter mean right front stride lengths compared to calves in the low lung lesion category (112 and 128 cm, respectively; *P* < 0.01).

Right front force did not differ by treatment by time interaction (*P* = 0.87) but did differ by treatment (*P* = 0.01), timepoint (*P* = 0.01), and lung category (*P* = 0.01). BRD + PLBO calves had significantly lower mean right front force (85.5 kg) compared with BRD + FTD calves (96.5 kg; *P* = 0.03). Calves had significantly lower mean right front force at 144 h (82 kg) relative to 32 h (96.9 kg; *P* = 0.04). Calves in the high lung lesion category had significantly lower mean right front force (80.0 kg) compared with calves in the medium and low lung lesion categories (91.3 and 95.5 kg, respectively; *P* < 0.03).

**Figure 2.**
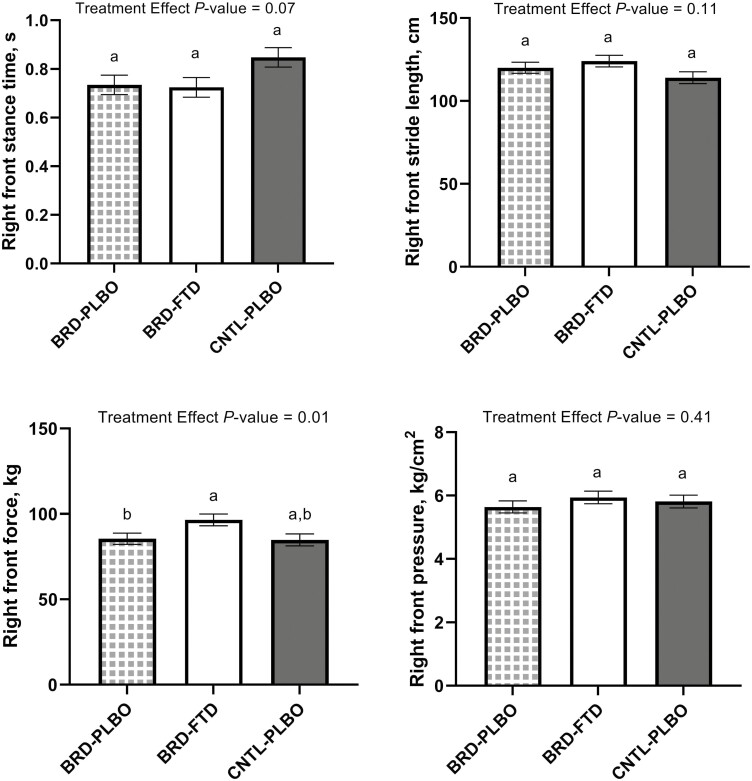
Mean values ± SEM for right front gait analysis outcomes by treatment. ^a,b^Different superscripts indicate significant differences between treatment groups (*P* ≤ 0.05).

Right front impulse did not differ by treatment (*P* = 0.22), timepoint (*P* = 0.11), treatment by time interaction (*P* = 0.66) or lung category (*P* = 0.20). Right front pressure did not differ by treatment (*P* = 0.41), treatment by time interaction (*P* = 0.59), or lung category (*P* = 0.16) but did differ by timepoint (*P* = 0.01). At the 32 h timepoint, right front pressure was the least (5.23 kg/cm^2^) and at 8 h it was the greatest (6.37 kg/cm^2^) but did not differ significantly. Average right front limb gait analysis outcomes are described in Figure 2.

**Figure 3.**
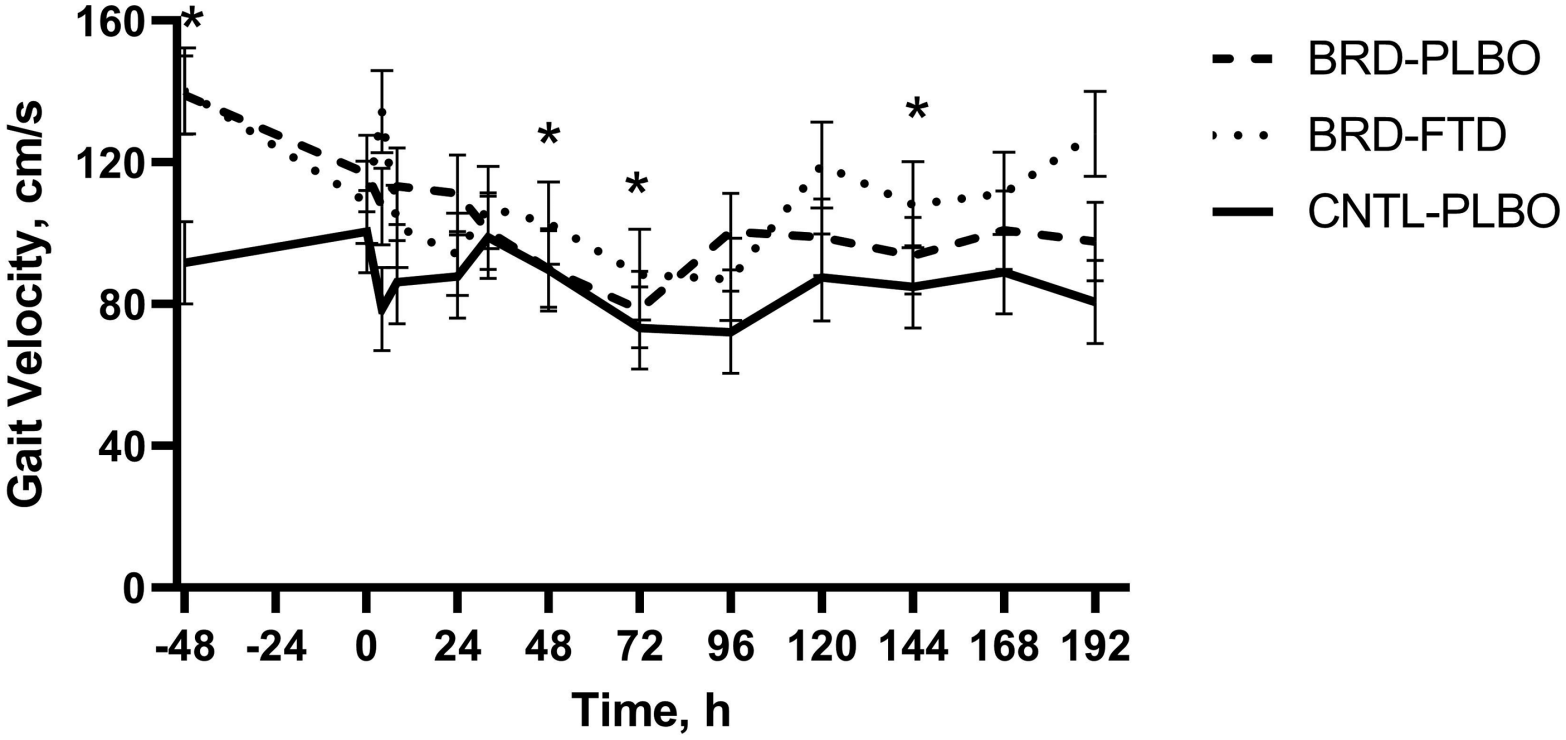
Mean values ± SEM for gait velocity over time by treatment group. ∗Timepoints where a statistically significant difference (*P* ≤ 0.05) was observed.

Gait distance did not differ by treatment (*P* = 0.86), timepoint (*P* = 0.06), treatment by time interaction (*P* = 0.32), or lung category (*P* = 0.23). Gait velocity did not differ by lung category (*P* = 0.07) but differed by a treatment by time interaction (*P* = 0.05; Figure 3). The BRD + PLBO group had greater velocity at −48 h (139 cm/s) compared with 48, 72, and 144 h (89.9, 78.5, and 93.6 cm/s, respectively; *P* < 0.05).

### Mechanical nociceptive threshold

Average MNT did not differ by treatment (*P* = 0.35), treatment by time interaction (*P* = 0.14) or lung category (*P* = 0.96) but did differ by timepoint (*P* < 0.01). Baseline average MNT means at −48 h (2.00 kg F) were larger than average MNT values at 24, 72, and 192 h (1.59, 1.66, and 1.23 kg F, respectively; *P* < 0.03). The percent change from baseline MNT values did not differ by treatment (*P* = 0.73), timepoint (*P* = 0.61), treatment by time interaction (*P* = 0.68), or lung category (*P* = 0.84).

### Visual analog scale

**Figure 4.**
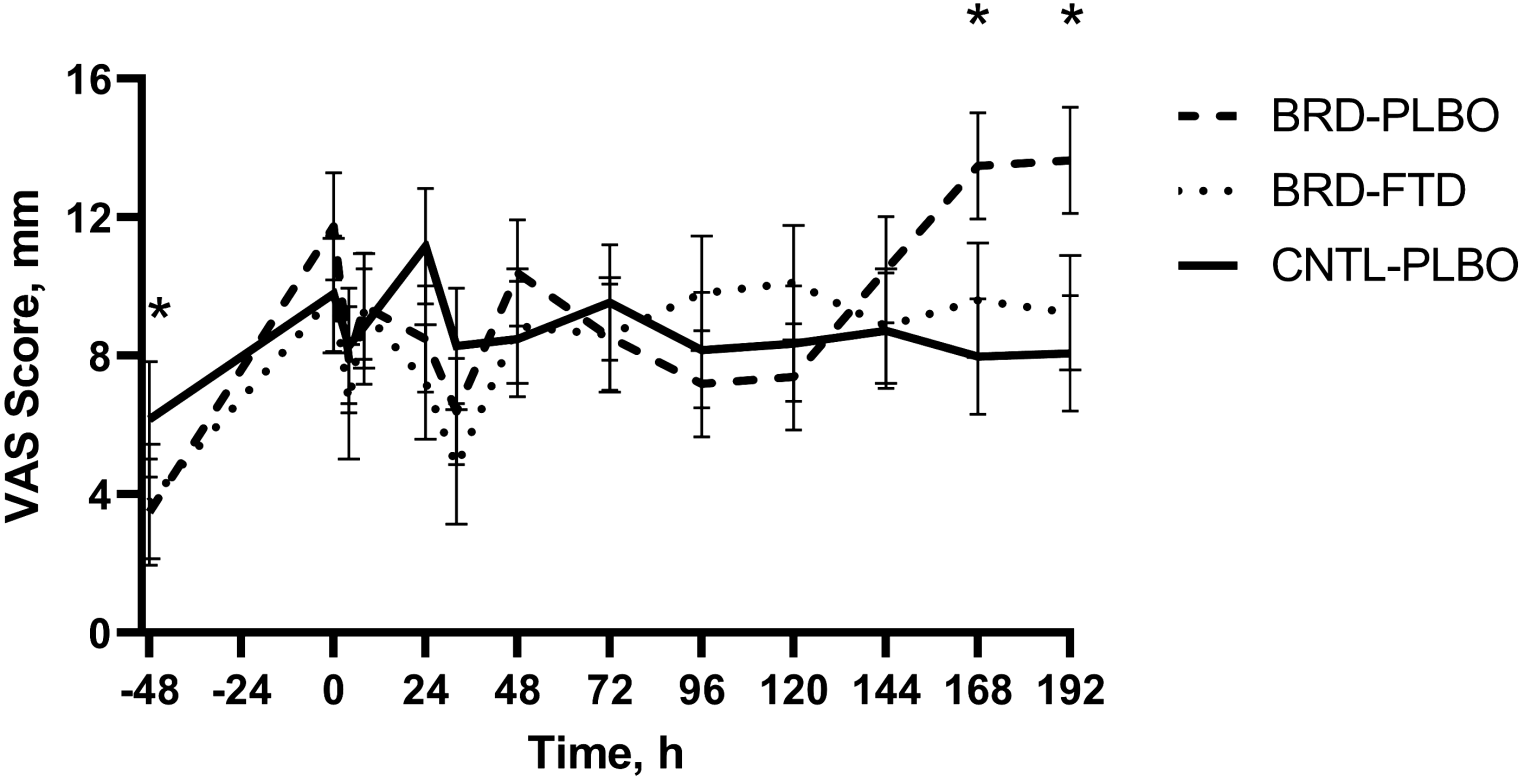
Mean values ± SEM for VAS over time by treatment group. ∗Timepoints where a statistically significant difference (*P* ≤ 0.05) was observed.

Average VAS scores did not differ by lung category (*P* = 0.07) but differed by treatment by time interaction (*P* = 0.01; Figure 4). The mean VAS scores differed for the BRD + PLBO group at −48 (3.49 mm) compared with 168 and 192 h (13.49 and 13.64 mm, respectively; *P* < 0.01).

### Clinical illness score

Clinical illness score did not differ by treatment (*P* = 0.96), treatment by time interaction (*P* = 0.57), or lung score (*P* = 0.10) but did differ by timepoint (*P* < 0.01). The probability of a CIS >1 at 4 h (0.09) was less than the probability of CIS >1 at 120, 168, and 192 h (0.67, 0.87, and 0.91, respectively; *P* < 0.03).

### Computerized lung score

Computerized lung score (**CLS**) did not differ by treatment (*P* = 0.84) or treatment by time interaction (*P* = 0.98) but did differ by timepoint (*P* < 0.01) and lung category (*P* = 0.05). Probabilities for calves having a CLS >1 at 8 and 24 h (0.17 and 0.20, respectively) were significantly less than at 32, 72, 96, 144, and 192 h (0.83, 0.83, 0.69, 0.78, and 0.83, respectively; *P* < 0.05). The probability of calves having a CLS >1 was significantly higher for calves in the high lung lesion category (0.73) compared with the medium lung lesion category (0.50; *P* = 0.05), there was not a difference between the high and low lung lesion categories (*P* = 0.23).

### Accelerometers

**Figure 5.**
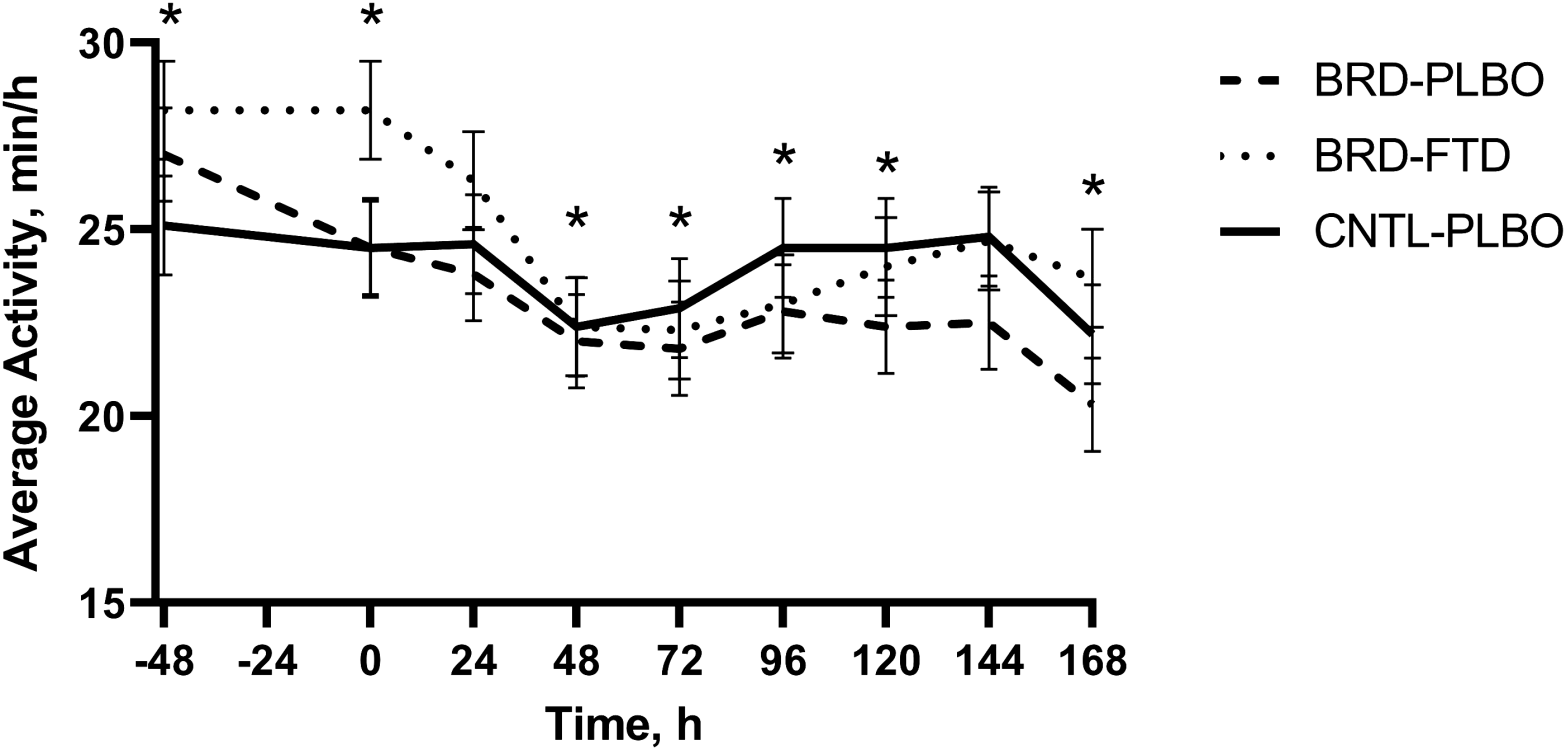
Mean values ± SEM for average activity levels over time by treatment group. ∗Timepoints where a statistically significant difference (*P* ≤ 0.05) was observed.

Average activity differed by treatment by time interaction (*P* = 0.01; Figure 5) and lung category (*P* = 0.01). Average activity for the BRD + PLBO group was higher at −48 h (27 min) compared with 48, 72, 120, and 168 h (≤ 22.24 min/h; *P* < 0.01). Average activity for the BRD + FTD group was significantly higher at −48 and 0 h (28.2 and 28.2 min/h, respectively) compared 48, 72, 96, and 168 h (≤ 23.7 min/h; *P* < 0.01). Average activity was significantly less in the high lung lesion category (20.9 min/h) compared with the low (27.2 min/h; *P* < 0.01).

Rumination activity did not differ by treatment (*P* = 0.73) or treatment by time interaction (*P* = 0.50) but did differ by timepoint (*P* = 0.01) and lung category (*P* = 0.01). Rumination activity at 96 h (41.5 min/h) was higher compared with 48 h (31.1 min/h; *P* < 0.04). Calves in the high lung lesion category had lower rumination activity levels compared with calves in the low lung lesion category (28.4 and 43.8 min/h, respectively; *P* < 0.04).

Motion index differed by timepoint (*P* < 0.01) and lung category (*P* = 0.01). Motion index at −48 and 0 h (6,983 and 7,195) was greater compared with 48 to 168 h (≤5,887; *P* < 0.01). Calves with high and medium lung lesion scores had a lower motion index (4,246 and 4,727, respectively) relative to calves with a low score (7,256; *P* < 0.01).

Standing time did not differ by treatment (*P* = 0.52), treatment by time interaction (*P* = 0.95), or lung category (*P* = 0.23) but did differ by timepoint (*P* < 0.01). Calves spent more time standing and less time lying at 0, 24, and 120 h (0.48, 0.50, and 0.52, respectively) compared with −48, 48, 72, 96, and 168 h (≤ 0.38; *P* < 0.01).

Step count differed by timepoint (*P* < 0.01) and lung category (*P* = 0.01). Calves took more steps at −48, 0, and 24 h (1,358, 1,544, and 1,350 steps, respectively) compared with 48, 72, 96, 144, and 168 h (≤1,045 steps; *P* < 0.01). Calves in the medium and high lung lesion categories took fewer steps (1,009 and 924 steps, respectively) than calves in the low category (1,471 steps; *P* < 0.02).

Lying bouts differed by timepoint (*P* = 0.01) and lung category (*P* = 0.02). Calves took more lying bouts at −48 and 72 h (14.7 and 14.4 bouts, respectively) relative to 144 h (11.2 bouts; *P* < 0.03). Calves in the high lung lesion category had fewer lying bouts (10.3 bouts) than calves in the low category (15.1 bouts; *P* < 0.04), there was not a difference between the high and medium lung lesion categories (*P* = 0.10).

### Rectal temperature

Average rectal temperature did not differ by treatment (*P* = 0.98), treatment by time interaction (*P* = 0.15) or lung category (*P* = 0.20) but did differ by timepoint (*P* < 0.01). Average rectal temperature at 120, 144, and 168 h (38.7, 38.6, and 38.6 °C, respectively) was lower than at 0, 8, 24, and 32 h (39.4, 39.7, 39.5, and 39.8 °C, respectively; *P* < 0.04).

### Cortisol

Cortisol concentrations did not differ by treatment (*P* = 0.19), treatment by time interaction (*P* = 0.10), or lung category (*P* = 0.76) but did differ by timepoint (*P* = 0.01). Cortisol levels were higher at −48 h (7.19 ng/mL) compared with 72, 144, and 168 h (2.41, 2.33, and 1.77 ng/mL, respectively; *P* < 0.04).

### Substance P

Substance P concentrations did not differ by treatment (*P* = 0.14), timepoint (*P* = 0.07) treatment by time interaction (*P* = 0.90), or lung category (*P* = 0.99).

### Prostaglandin E_2_ metabolite

PGEM concentration differed by timepoint (*P* < 0.01) and lung category (*P* = 0.01). Concentrations of PGEM were lower at 72 and 192 h (20.8 and 21.2 pg/mL, respectively) relative to −48 and 0 h (36.0 and 38.8 pg/mL, respectively; *P* < 0.01). Concentrations of PGEM were lower in the low lung lesion category (18.5 pg/mL) relative to the high lung lesion category (39.1 pg/mL; *P* < 0.01).

### Serum amyloid A

Concentrations of SAA did not differ by treatment (*P* = 0.59), treatment by time interaction (*P* = 0.24) or lung category (*P* = 0.44) but did differ by timepoint (*P* < 0.01). Concentrations of SAA were higher at 4, 8, 24, and 32 h (310, 366, 369, and 323 µg/mL) compared to −48 and 120 to 192 h (≤175 µg/mL; *P* < 0.03).

### Lung lesion score

Lung lesion scores ranged from 1% to 34%. In the control group, 37.5% of the calves received a lung lesion score >10%. Of the calves inoculated with *M. haemolytica*, 87.5% of the calves that received flunixin transdermal received a lung lesion score >10%, and 90% of the untreated calves who received a placebo had a lung lesion score >10%. About 73% of the calves on study had a medium or high lung lesion score. Lung lesion score differed by treatment (*P* = 0.03). Calves in the CON group had lower lung lesion scores (10.3) than BRD + PLBO calves (20.4; *P* = 0.05).

### Plasma flunixin concentrations

**Figure 6.**
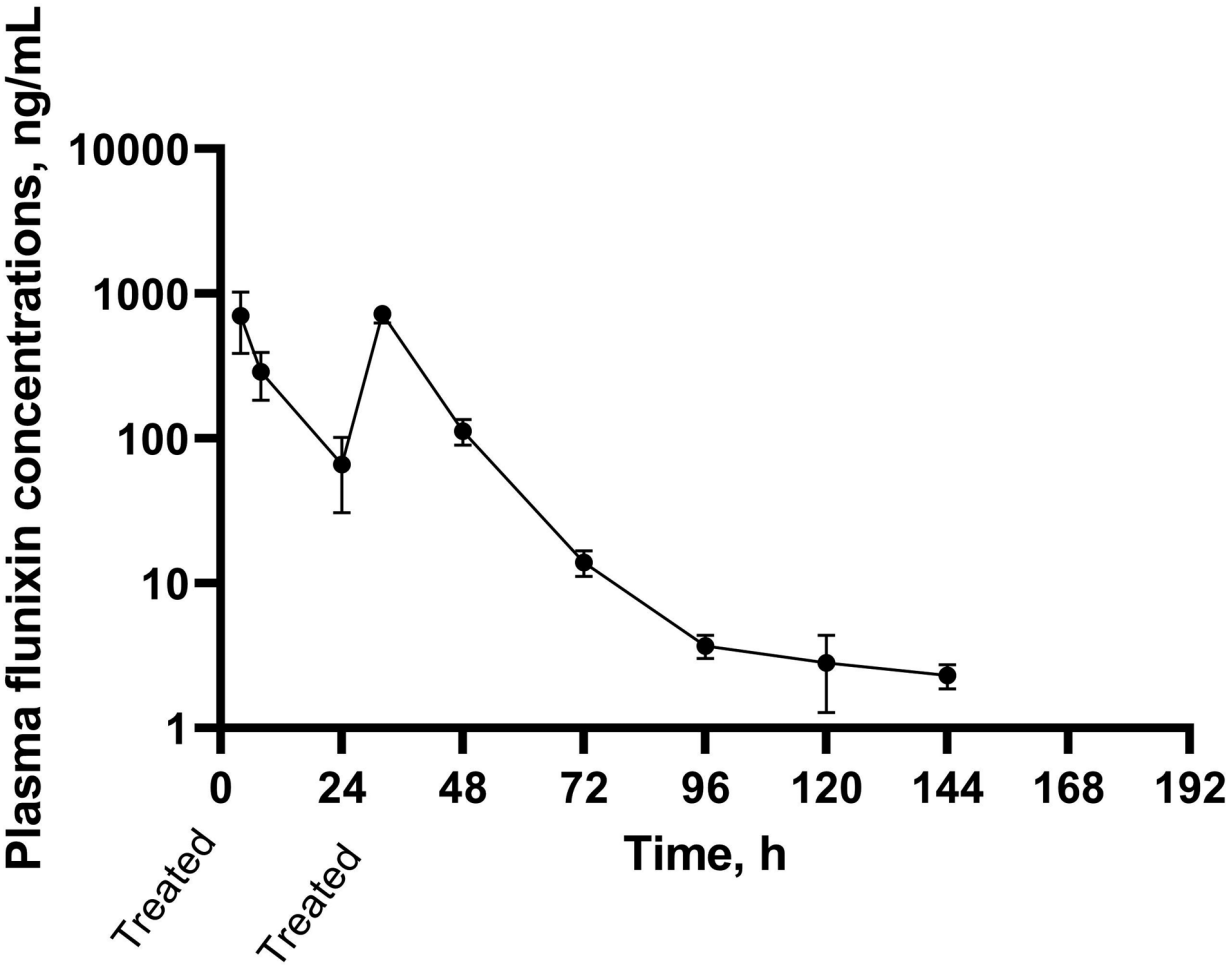
Arithmetic mean values ± SEM for plasma flunixin concentrations on a log scale over time in the BRD + FTD treatment group.

Mean plasma flunixin concentrations are outlined in Figure 6. The limit of detection and limit of quantification for the plasma flunixin analysis were 1.0 and 1.46 ng/mL, respectively. The mean *λ*_z_ was 0.058 1/h, T½ was 11.18 h, C_max_ was 238.05 ng/mL, T_max_ was 4.27 h, AUC_0–last_ was 16,943.29 (h∗ng/mL), and AUC_0–∞_ was 16,953.53(h∗ng/mL). The range for T½ was 9.54 to 24.06 h and for C_max_ was 29.23 to 2,590.37 ng/mL.

### Correlation coefficients

**Figure 7.**
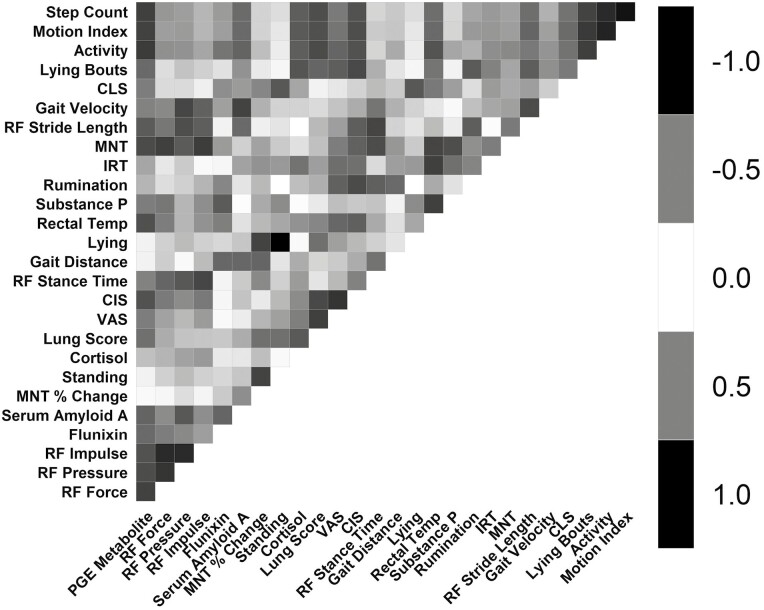
Pearson correlation coefficient for each outcome parameter collected.

Pearson correlation coefficients are shown as a heatmap in Figure 7. VAS score showed evidence of being positively correlated with CIS (Pearson correlation coefficient: 0.69). Gait velocity showed evidence of being negatively correlated with right front stance time (Pearson correlation coefficients: −0.74 and −0.68, respectively) and positively correlated with right front stride length (Pearson correlation coefficient: 0.63). Average activity showed evidence of being positively correlated with motion index and step count (Pearson correlation coefficients: 0.78, 0.75, respectively).

Lung lesion score and activity average showed evidence of being negatively correlated at 48, 72, 96, 120, 144, and 168 h (Pearson correlation coefficients: −0.67, −0.69, −0.72, −0.66, −0.71, and −0.69, respectively). Lung lesion score and step count showed evidence of being negatively correlated at 72 and 96 h (Pearson correlation coefficients: −0.62, −0.63). Lung lesion score and rumination average showed evidence of being negatively correlated at 168 h (Pearson correlation coefficient: −0.64).

## Discussion

Pleuritic chest pain resulting from bacterial pneumonia is commonly reported in human medicine (Boyd et al., 2006). Percussion of the thoracic wall has been reported to elicit signs of pain and painful respiration has been documented from pleuropneumonia in cattle, with these findings published over 20 yr ago (Ter Laak, 1992; Braun et al., 1997). Whether or not cattle experience some form of pleuritic chest pain from bacterial pneumonia is not well-characterized. The BRD induction model used in the present study was expected to produce bacterial pneumonia and gross necropsy findings revealed pleuritis was present in inoculated calves with significant lung consolidation. The evaluation of pain in cattle requires a multifaceted approach as there is no single biomarker for pain, which may contribute to the lack of literature regarding the association of pain and BRD. The results from the present study indicate that a combination of reduced activity levels, decreased force on calves’ right front limb near the area of bacterial infection, and increased VAS pain scores all support that pleuritis associated with bacterial pleuropneumonia in cattle is painful.

Gait analysis seemed to be the most objective measure that detected treatment differences that may be a good indicator of BRD and specific to pain. Calves who received flunixin transdermal placed more force on their right front limb compared to calves inoculated with *M. haemolytica* who did not receive an NSAID, providing evidence that flunixin may have attenuated pain. In the present study, the right apical lung lobes were inoculated, thus indicating that gait measurements more specific to the area of disease pathology may yield better results. For the study duration, right front force decreased as BRD progressed indicating that calves placing less force on their right front limb may be more painful. In a study examining gait following soft-tissue surgery in sheep, sheep who had undergone surgery placed less weight on their hind limbs compared with controls (Viscardi et al., 2021). The hind limbs were nearest the surgical site supporting the idea that animals may place less weight on limbs in close proximity to where the animal is experiencing pain.

VAS pain scores showed the most pronounced increase in calves inoculated with *M. haemolytica* who did not receive an NSAID over the duration of the study compared to other treatment groups. VAS scoring is a more subjective outcome but may be more sensitive to pain relative to CIS based on treatment differences being detected by VAS that were not detected with CIS. This may be due to CIS being designed to assess the severity of undifferentiated respiratory disease by signs of depression, lowered head, and ability to rise (Perino and Apley, 1998), whereas the VAS used quantified depression, stance, head carriage, spinal alignment, movement, and ear carriage (Martin et al., 2020) which may have captured more specific pain behaviors identified by Gleerup et al. (2015).

Activity levels significantly decreased for both treatment groups inoculated with *M. haemolytica* relative to controls indicating that reduced activity levels may be a good indicator of BRD onset but less specific to pain, similar results were reported by Hanzlicek et al. (2010). Pillen et al. (2016) reported reduced activity levels at least 6 d prior to BRD diagnosis with more pronounced changes the day before disease identification. Sick animals often increase time of rest to reduce energy expenditure (Hart, 1988) and remotely quantifying activity levels may become a useful way to identify sick cattle earlier on in the disease process before more clinical signs manifest themselves.

Lung lesion scores were assigned by a pathologist (K.M.A.) at the K-State Veterinary Diagnostic Lab following the 192 h sample collection and euthanasia. Calves with a high degree of lung consolidation were likely experiencing more thoracic pain than calves with very little lung consolidation; however, at what point this consolidation occurred throughout the disease process and duration of the study for each animal is not known. Calves in the control group had lower lung lesion scores than untreated calves inoculated with *M. haemolytica*. Human reports of pleuritic chest pain followed up with examination and chest X-ray have revealed areas of consolidation (Boyd et al., 2006) providing a link between pain and lung consolidation. High lung lesion scores were associated with a lower mean right front stride length and force, CLS >1, lower average activity and rumination levels, lower motion index and step count, fewer lying bouts, and higher PGEM concentrations. Previous literature has found distance traveled (White et al., 2012) and CLS (Baruch et al., 2019) to be associated with lung lesion scores. Previous studies have shown changes in biomarker outcomes predictive of BRD include salivary cortisol and IRT (Schaefer et al., 2007), SAA (El-Deeb et al., 2020), activity and rumination (Marchesini et al., 2018), and accelerometer activity (Belaid et al., 2019).

This was an exploratory study investigating a large number of potential biomarkers to assess pain associated with BRD. CLS has been found to have relatively high sensitivity and specificity (92.9% and 89.6%, respectively) when diagnosing BRD compared with pen checking (Mang et al., 2015). Median sensitivity for CIS has been found to range from 82% to 99% and specificity from 81% to 95% with sensitivity increasing and specificity decreasing as lung consolidation scores increased (Amrine et al., 2013). Sensitivity and specificity for ear tag mounted accelerometers has been found to be 95% and 76% for feeding and 49% and 96% for rumination, respectively (Wolfger et al., 2015). The diagnostic accuracy of the biomarker and clinical sign collection methods employed in the current study likely impacted the internal validity of the study. The authors chose a challenge study design to measure biomarker outcomes at similar stages of disease progression in a cohort without the confounders introduced by waiting for naturally occurring cases to present themselves that could have resulted in varying clinical signs and disease severity. Synthesizing results from the current study and using them to make an informed decision of what biomarker outcomes to collect in a naturally occurring BRD model would greatly add to the external validity of literature quantifying pain from BRD. A post hoc power calculation was completed for substance P due to an observed numerical difference and trend towards significance in the *P*-value. A sample size of eleven calves per treatment group was determined based upon an effect size of 0.576, indicating that a larger sample size may have yielded different results for secondary outcomes that the original sample size was not based upon.

This study was conducted in late fall with changing weather and large temperature swings that created conditions likely to produce respiratory disease outbreaks in calves. All the calves on study received thoracic ultrasounds and had not shown signs of respiratory disease prior to being on study and were not allowed to touch noses with other calves on site. However, inoculating the control calves with saline via bronchoalveolar endoscopy seemed to create enough of a stressful event in the described conditions that the control calves may have experienced some degree of pain and distress, as well as the calves inoculated with *M. haemolytica*. Theurer et al. (2013) employed a similar method of inoculating controls with saline but the study occurred a different time of year when high ambient temperatures were recorded. The percentage of calves in the control group with a >10% lung lesion score likely influenced treatment differences and thus the authors will discuss overall changes in outcomes across treatment groups due to 73% of the calves on study having medium or high lung lesion scores. Removing calves in the control group with >10% lung lesion scores from the analysis was investigated but caused the analysis to be underpowered and did not reveal different study findings.

Throughout the duration of the study, the probability of CIS >1 gradually increased from baseline. Baruch et al. (2019) had similar results with baseline CIS scores differing from those during the bacterial phase of BRD infection. Mechanical nociceptive threshold (**MNT**) values, right front stride length, substance P concentrations, motion index, and step count all decreased from baseline over the study duration. Williams et al. (2020) introduced using MNT in the thoracic region in cattle and established the location used in the present study as repeatable and feasible. Sensitivity to force in the thoracic region is the most tangibly direct measure of chest pain used in the present study and may have potential as an indicator of thoracic pain from BRD. SAA values sharply increased at 0 h, plateaued and began decreasing towards baseline values at 72 h. These results agree with the findings of Petersen et al. (2004) that an elevation in SAA is observed in cattle with viral as well as bacterial respiratory infection. Cortisol values were very low throughout the duration of the present study. Theurer et al. (2013) found an increase in cortisol concentrations following *M. haemolytica* challenge, but Foote et al. (2017) did not find differences in cortisol due to BRD classification. The calves in the current study were well acclimated to human interaction and moving through the handling facility leading us to believe that the cortisol response to restraint may have been mitigated, and in this study, there was no clear cortisol response to *M. haemolytica* challenge.

During the treatment administration at 0 h, heavy rainfall occurred, and thus the authors chose to readminister the flunixin transdermal and placebo treatment at 24 h. A previous study has shown that rainfall immediately after treatment reduces flunixin transdermal absorption (Altenbrunner-Martinek et al., 2020). While the objective of this study was not to describe flunixin transdermal pharmacokinetics (**PK**), the authors chose to outline the PK parameters following the first dose of flunixin transdermal administration due to the impacts of rainfall and the results were closely aligned with those of Altenbrunner-Martinek et al. (2020). The T_max_ for flunixin transdermal has been shown to be 2.14 h (Kleinhenz et al., 2016) and samples were not collected until 4 h following flunixin transdermal administration in the present study, thus impacting the T_max_ and C_max_. The mean half-life of flunixin transdermal has been found to be 6.42 h by Kleinhenz et al. (2016) and 8–9 h in the PK studies conducted for Food and Drug Administration (**FDA**) approval (2017) but was longer in the present study at 11.18 h. This longer half-life may have been due to the disease state or age of the animals (Leavens et al., 2014) or the number of observations considering a wide range in values was recorded.

The study duration was 192 h in order to capture the evolution of the BRD disease process over a series of days. This was a similar time period investigated by (Theurer et al., 2013). Hanzlicek et al. (2010) necropsied calves following inoculation from day 1 to 9 and found gross lung lesions from day 1 to 3 with resolving pneumonia along with atelectasis and some dense fibrin found in lungs harvested day 5 to 9. The authors did not want to cause unnecessary pain and suffering by prolonging disease progression beyond 192 h. The results from the current study indicate that many pain outcomes had not returned to baseline levels at the end of the study, pointing to the need for long-acting analgesic therapies to alleviate pain from BRD. Part of the analgesic approval process requires effectiveness studies in which clinical endpoints must be chosen (Smith, 2019). Therefore, correlations between biomarkers become important when choosing which endpoints to evaluate. Correlations in the present study were strong between VAS score and CIS, both of which are subjective measures evaluated by a trained observer. Lung lesion scores were negatively correlated with activity levels from 48 h to the end of the study, with step count from 72 to 96 h and with rumination levels at the end of the study period. Further investigation into biomarker correlations and which combination of biomarkers is most appropriate for quantifying pain from BRD is warranted.

## Conclusions

The results from the present study indicate that a combination of reduced activity levels, decreased force on calves’ right front limb, and increased VAS pain scores all support that bacterial pneumonia in cattle is painful. Differences in right front force were observed in calves challenged with *M. haemolytica* and treated with flunixin transdermal and those given a placebo, indicating that flunixin transdermal may attenuate specific pain biomarkers in cattle with BRD. Lung lesion scores were negatively correlated with activity levels from 48 h to the end of the study, with step count from 72 to 96 h and with rumination levels at the end of the study period. Investigating biomarkers associated with pain from BRD and its alleviation with analgesic compounds warrants further research to prevent pain and suffering in cattle.

## Abbreviations

BRD: bovine respiratory disease
CIS: clinical illness score
CLS: computerized lung score
CNTL: control
EDTA: ethylenediaminetetraacetic acid
FTD: flunixin meglumine transdermal
IACUC: Institutional animal care and use committee
IBC: institutional biosafety committee
IRT: infrared thermography
MARC: Meat Animal Research Facility
MNT: mechanical nociceptive threshold
NSAID: nonsteroidal anti-inflammatory drug
PGEM: prostaglandin E_2_ metabolite
PLBO: placebo
SP: substance P
USDA: United States Department of Agriculture
VAS: visual analog scale
